# 

**Published:** 1860-04

**Authors:** 


### CONTENTS.

### APRIL, 1860.

### Lectures, Monographs, and Cases.

PAGE

On Plastic Operations. By B. Langenbeck, Prof. of Surgery in the University of Berlin. Translated and Communicated by William F. Holcomb, M.D. .... 657

Lectures on Displacements of the Uterus. By E. R. Peaslee, M.D., LL.D., Prof. of Obstetrics and Diseases of Women and Children in the New York Medical College. Lecture II .... 668

On the Mechanical Means adopted in the Treatment of Morbus Coxarius. By H. G. Davis, M.D. (With a Plate.) .... 678

A Glance at Medical Science among some of the East Indian Nations. From the French of Dr. L. S. Heymann .... 684

Dragées of Iron, Manna, and Bismuth. By Dr. Morin .... 690

Action of Chloride of Zinc as a Caustic. By Salmon and Maunoury .... 692

Spontaneous Cure of Cancer of the Breast .... 693

Topical Application for Tumors of the Breast .... 693

Alum Pastilles .... 694

Glucosuria in Paludal Fever .... 694

### Monthly Summary of Medical Journalism,

By O. C. Gibbs, M.D., Frewsburg, N. Y.

Bloodletting in Inflammation, &c., 695; Nitric Acid in Remittent Fever of Adynamic Type, 697; Vesico-Vaginal Fistula, 697; Hydrophobia, 699; Diphtheria. 699; Tobacco in Rattlesnake Bites, 701; Endermic Medication, 702; Stomatitis Materna, 702; Strychnia in Seminal Emissions, 703; Wound of the Brain, 703; Tongue Removed by the Ecraseur, 703; Radical Cure of Hernia, 703–4; Twelve Successful Operations by Wurtzer's Method, 705; Induction of Premature Labor, 705; Influence of the Mother's Mind on the Fœtus in Utero, 706; Transfusion, 706; Chronic Rheumatism, 707; Water Dressings, 708; Scarlet Fever, 709; Asthma, 709; Treatment of Epilepsy, 710; Dysentery, 710; Scarlatina, 711.

### Proceedings of Societies.

N. Y. Medico Chirurgical College—Discussion on Morbus Coxarius .... 712

### Editorial and Miscellaneous.

Osteo-Plastic Operations—Letter from Dr. Holcomb—Blood as Food—Organoscope—Commencements in N. Y. Medical Schools—Resignation and Appointment in the University of Maryland—Death of Prof. Charles Frick—Specialties in Medicine and the Paris Faculty—Suicides in Sweden—A Medical Astronomer—Hydrochloric Acid in Small-Pox—Pencils of Tannin in Affections of the Uterus—Unguentum Glycerini, &c., &c. —Dr. John O'Reilly's Prizes—Books and Pamphlets Received .... 726

